# How protein kinase inhibitors bind to the hinge region of the target protein.

**DOI:** 10.1063/4.0000906

**Published:** 2025-10-27

**Authors:** Urszula Derewenda, Steve Scheiner, Zygmunt S Derewenda

**Affiliations:** 1 University of Virginia, Charlottesville, Virginia, USA; 2 State University of Utah, Logan, Utah, USA

## Abstract

The human genome encodes 518 protein kinases which make up one of the most important families of regulatory proteins, catalyzing phosphorylation of hydroxyl-containing amino acids, i.e. tyrosine, serine and threonine [1]. These enzymes are involved, among others, in the regulation of cell cycle [2], cell growth and proliferation, as well as inflammation an immunological processes [3]. All of these phenomena are of significance in tumorigenesis and tumor growth and metastasis, and protein kinases are of-ten selectively overexpressed in a number of cancer types [4]. As a result, these regulatory enzymes now constitute one of the most important families of anti-cancer and immuno-logical disorder drug targets [3, 5-12]. As of January 2024, there were 80 protein kinase inhibitors approved by the FDA for clinical use, including 69 used to treat cancer an non-malignant neoplasm, and six for the treatment of inflammatory diseases [11]. More than 700 other protein kinase inhibitors are in clinical trials, promising to expand this class of drugs [13]. However, at this moment only ∼100 kinases are targeted, while the function of the remaining ones, and their potential as drug targets, are yet to be elucidated [14].

Protein kinases are typically large, multi-domain proteins, and the catalytic activity is harbored within a specific enzymatic module, which show significant amino acid and structural conservation, particularly within the ATP-binding pocket, which is targeted by most inhibitors [1, 15, 16]. The kinase catalytic domain is made up of two ‘lobes’: the N-terminal lobe (N-lobe), which is structurally highly malleable and subject to regulation, including phosphorylation, and the stable, α-helical C-lobe which is the substrate binding site [17]. The large solvent-accessible cavity between the two lobes is the binding site for ATP, organized in such a way that the adenine moiety penetrates most deeply, and is recognized via hydrogen bonds by a short stretch of the polypeptide chain linking the two lobes, i.e. the hinge motif [18]. Kinase inhibitors belong to six distinct types [11]. Types I and II, which include the vast majority clinical drugs, occupy the ATP-site and are therefore ATP competitors [19]. Type III and IV bind to other sites, one of which is a regulatory site adjacent to the ATP pocket and the other an allosteric site. Type V are bivalent inhibitors which may use the ATP site, while type VI are covalent inhibitors, that attach to a cysteine (or sometimes ly-sine) residues close to the ATP binding site, with an active module of the compound blocking irreversibly the latter. Thus, most clinically used inhibitors contain a module that acts as an anchor binding to the hinge motif.

The ATP-pocket of kinases is selective for adenine, vs guanine, owing to a specific hydrogen bond (H-bond) network, involving the hinge. Three amino acids that make up the hinge are in extended, β-sheet conformation, denoted gk+1, gk+2 and gk+3 in accordance to their position downstream in the sequence from the ‘gatekeeper’ residue (or gk) [20]. It was first noted in 1997 [21] that a number of ATP-binding proteins share a main chain architecture found in kinases, that facilitates H-bonding of the gk+1 carbonyl to the 6-amino group, and the gk+3 peptide amide to N1 of adenine [22]. Subsequently, it was noted that many kinase inhibitors show a similar pattern of H-bonding, engaging the same main groups of the hinge, and thus mimicking ATP [18]. Recently, a structural database of 2,705 complexes of kinase domains with inhibitors was analyzed and helped to identify fifteen distinct binding modes [23]. Such studies are valuable for the design of alternative hinge binding motifs to assist in structure based drug design [24] (FIGURE 1).

The above-cited studies focused on the canonical H-bonds between electronegative atoms in both inhibitors and enzymes. In reality, kinase- inhibitor interactions are mediated also by a plethora of C-H…O bonds, which—while weaker—have the potential to make significant contribution [25-28]. The existence of these bonds in proteins and protein-ligand interactions, as well as their energies, are well established [25, 29, 30]. In fact, adenine forms not two, but three H-bonds, with the third one mediated by the C2-H group of adenine which donates a proton to the main chain carbonyl of gk+3 (FIGURE 2). The presence of such bonds at kinase-inhibitor interfaces has been recognized for a long time [31, 32]. We have recently shown by mining the Protein Data Base, that the vast majority of kinase inhibitor scaffolds contain a specific core moiety—typically of an aromatic nature—which mimics the interaction of adenine with the hinge with three H-bonds, including canonical and C-H…O [33]. These core moieties contain H-bond donors (including methine groups, i.e. =CH-) and acceptors, which interact with the gk+1 and gk+3 carbonyl groups and the gk+3 amide [33]. Specifically, we identified three distinct templates where one or two of the potential H-bond sites involve a C-H donor from the inhibitor. However, our studies were limited to the analyses of the stereochemisty derived from crystal structures. Although as the presence of cohesive H-bonds can be inferred with reasonable accuracy from interatomic distances and angles, they fail to provide insight into energetic contributions, and therefore to the function and significance of specific interatomic interactions. It is also not clear, for example, if the core-hinge interaction is a major determinant of the binding affinity (or kD), or if such interactions are optimal in specific cases, or if are hindered by secondary enzyme-inhibitor contacts. Dissection of these questions is of paramount importance to open new approaches in structure-based drug design. To address these important questions, we resorted to quantum mechanics (QM) as implemented in Gaussian 16 [34] and Atoms-In- Molecules (AIM) [35], and the available X-ray crystallographic structures of a number of FDA-approved kinase-inhibitor complexes. We analyzed representative complexes and in each case dissected the energetic contribution of each of the H-bonds involved. We show that the energies of interactions of the cores of inhibitors and their target hinge motifs is mostly dependent on the three H-bonds, of which typically at least one is of the C-H…O type. Importantly, the outcome of the calculations is highly dependent on the quality of the crystallographic model, as well as the intrinsic limitations of each of the computational methods. Our results should are of importance to drug design/discovery projects.
